# Projecting the impact of climate change on honey bee plant habitat distribution in Northern Ethiopia

**DOI:** 10.1038/s41598-024-66949-3

**Published:** 2024-07-09

**Authors:** Haftom Gebremedhn, Yikunoamlak Gebrewahid, Gebremedhin Gebremeskel Haile, Gebre Hadgu, Tesfay Atsbha, Teweldemedhn Gebretinsae Hailu, Gebreamlak Bezabih

**Affiliations:** 1 Ghent University, Ghent, Belgium; 2 Tigray Agricultural Research Institute, Mekelle, Ethiopia; 3 Department of Earth and Environmental Sciences, Wesleyan University, Middletown, USA; 4 Institute of Animal Science, University of Hohenheim, Stuttgart, Germany; 5 Department of Animal Sciences, Aksum University, P. O. Box 314, Shire, Ethiopia

**Keywords:** Bee forage, Climate change, Habitat suitability, Honey bee, Zoology, Entomology, Climate-change impacts, Agroecology, Ecology, Biodiversity

## Abstract

Climate change significantly affects the diversity, growth, and survival of indigenous plant species thereby influencing the nutrition, health and productivity of honey bees (*Apis mellifera*). *Hypoestes forskaolii* (Vahl) is one of the major honey bee plant species in Ethiopia’s Tigray region. It is rich in pollen and nectar that typically provides white honey, which fetches a premium price in both local and inter-national markets. Despite its socio-economic and apicultural significance, the distribution of *H. forskaolii* has been declining, raising concerns regarding its conservation efforts. However, there is limited knowledge on how environmental and climatic factors affect its current distribution and response to future climate change. The study investigates the current and projected (the 2030s, 2050s, 2070s, and 2090s) habitat distributions of *H. forskaolii* under three future climate change scenarios (ssp126, ssp245, and ssp585) using the Maximum Entropy Model (MaxEnt). The results show that land use (50.1%), agro-ecology (28%), precipitation during the Driest Quarter (11.2%) and soil texture (6.1%) predominantly influence the distribution of *H. forskaolii,* collectively explaining 95.4% of the model's predictive power. Habitats rich in evergreen trees and mosaic herbaceous with good vegetation cover are identified as the most suitable for *H. forskaolii*. The spatial distribution of *H. forskaolii* is concentrated in the highlands and mid-highlands of the eastern and southern parts of Tigray, characterized by a colder temperature. Across the three climate change scenarios, the size of suitable habitat for *H. forskaolii* is projected to decrease over the four time periods studied. Predictions under the ssp585 scenario reveal alarming results, indicating a substantial decrease in the suitable habitat for *H. forskaolii* from 4.26% in the 2030s to 19.09% in the 2090s. Therefore, given the challenges posed by climate change, research efforts should focus on identifying and evaluating new technologies that can help the *H. forskaolii* species in adapting and mitigating the effects of climate change.

## Introduction

Beekeeping significantly contributes to food and nutrition security, socio-economic development, and ecosystem conservation through the pollination services of honey bees (*Apis mellifera*) and the production of valuable products including honey, beeswax and propolis worldwide^1–4^. The contribution of the beekeeping sector in Ethiopia is significant in income generation, agricultural productivity, environmental protection, and providing economic and employment opportunities to the population^4^. The country has diverse agroclimatic conditions that provide a conducive environment for sustaining over 6000 plant species, many of which are honey bee plants^5^. These favorable ecological conditions also enable the country to sustain an estimated 7 million managed honey bee colonies in addition to the numerous feral colonies^6^. The diversity of honey bee subspecies in Ethiopia has been subject of debate between different studies. Earlier classical morphometric analysis reported five honey bee subspecies in Ethiopia (*A. m. jemenitica, A. m. scutellata, A. m. monticola, A. m. bandasii, A. m. weyi-gambela*)^7^, but recent studies based on molecular, standard morphometric and geometric morphometric analyses designated all honey bees in Ethiopia as *A. m. simensis*^8–10^. With this potential, Ethiopia’s annual honey production reached around 130,000 tons in 2021, positioning it among the top ten honey-producing countries worldwide^6^.

The Tigray Regional State is one of the major beekeeping areas in Ethiopia. The sector plays a crucial role in creating jobs, generating income and food and nutritional security for more than 58,000 small-scale beekeepers and other rural communities^11–13^. This region is known for its white honey production, which is popular in Ethiopia’s domestic markets. Tigray’s white honey is often considered a premium product and the local beekeepers receive higher prices for their white honey due to its unique color, taste, flavor, aroma, quality and medicinal properties. The price of Tigray’s white honey is comparable to prices of honey in Europe, thus significantly contributing to the economic development and livelihood of smallholder beekeepers^5,14,15^.

The flavor, color, and various physical, chemical, and nutritional properties of honey are primarily influenced by its botanical origin and the climatic conditions of production areas^14–16^. Color, among the physical properties, is immediately perceived by consumers and plays a crucial role in the acceptance and valuation of honey^15^. *Hypoestes forskaolii* (Vahl), locally known as *Girbia*, is among the major honey bee plant species in Ethiopia that produce white honey^5,15^. Honey bees foraging on the plant for its abundant pollen and nectar^17–19^. In Tigray, *H. forskaolii* flowers from September to November, which highly synchronizes with the main honey flow period in the region^5,18,20,21^. The honey from this plant is creamy white and granulates easily, and was classified as mono floral honey in some areas of Tigray^5,15^. Due to its attractive colour and light taste, the white honey from *H. forskaolii* fetches a premium price in both local and international markets^5^. Local beekeepers and extension workers in Tigray regarded *H. forskaolii* as the best honey bee forage^18^. *H. forskaolii* is also used in traditional medicine in Ethiopia, Eritrea^22,23^ and Yemen^24^ to manage diabetes.

Despite its socio-economic and apicultural significance, the population of *H. forskaolii* is declining, which has threatened it in recent decades^25^. This decline may be attributed to various natural and anthropogenic pressures such as deforestation, frequent drought, and expansion of agricultural land. In addition, climate change has become a clear threat to the distribution, growth, and survival of many indigenous plant species particularly in recent years^26,27^. These may seriously affect honey bees by destroying crucial plant species such as *H. forskaolii* that serve as natural food sources^28^ and may lead to the species becoming geographically endangered. Therefore, understanding how these bee plants respond to global climate change is crucial for managing their habitat distribution and management strategies. However, knowledge on how local climate change influences the ecological distribution of *H. forskaolii* is limited. The cultivation of *H. forskaolii* also faces challenges due to the lack of knowledge availability of suitable habitats for the plant. Addressing these issues is crucial for planning restoration and conservation initiatives, as well as for reintroducing it into its suitable habitat that can prevent the extinction of the species and conserve its biodiversity. Therefore, this study was designed to model the current and future distribution of *H. forskaolii* under three shared socioeconomic pathway scenarios (ssp126, ssp245, and ssp585). The study was also aimed to determine the association of key environmental variables (including soil, land cover, topography, elevation, and 19 bioclimatic variables) with the distribution of *H. forskaolii*.

## Materials and methods

### Study area

**Figure 1:**
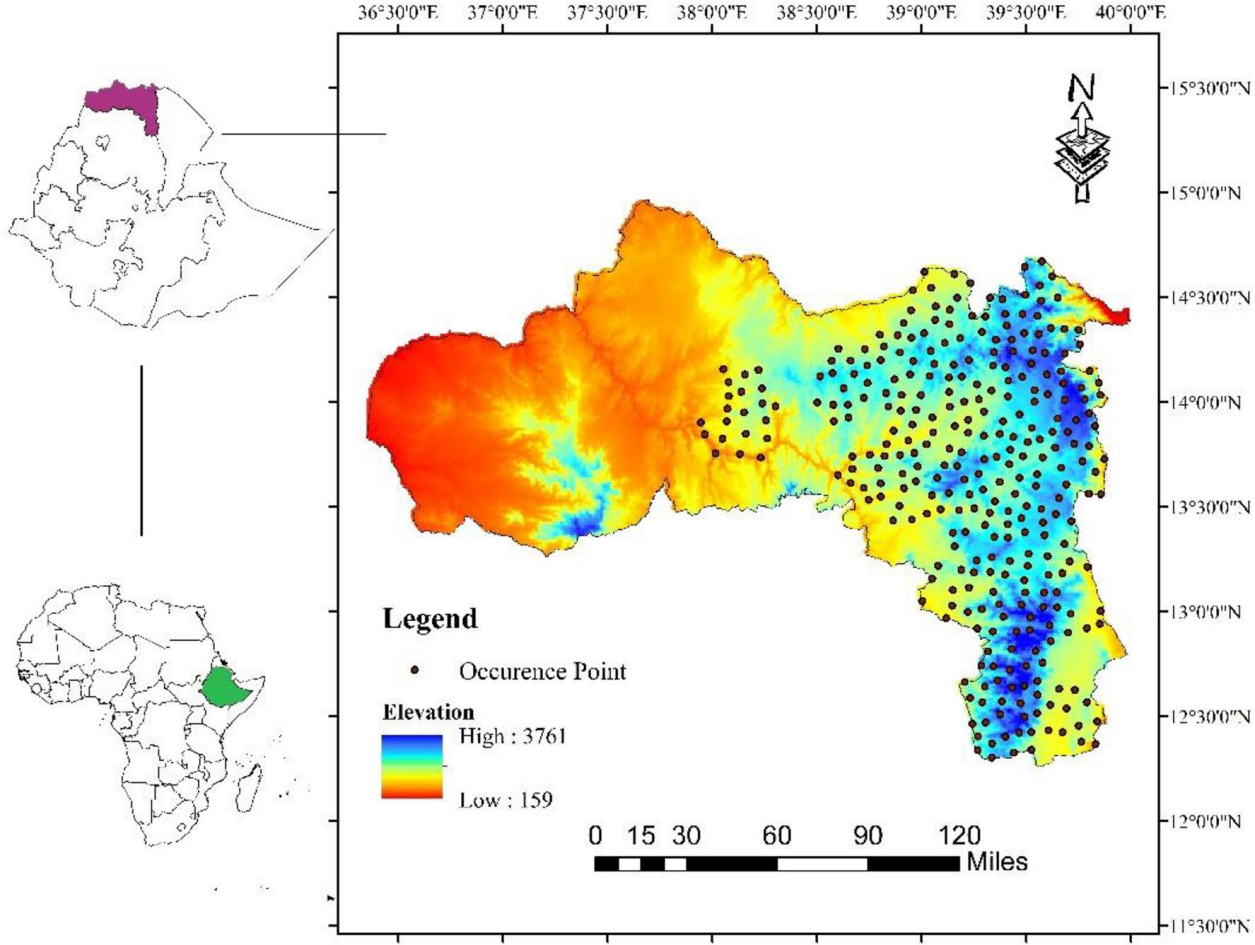
Maps showing the occurrence records of *H. forskaolii* in Tigray, Ethiopia. Figures were created using ArcMap (version 10.4).

Tigray is the north most regional state in Ethiopia (Fig. 1), sharing borders with Sudan, Eritrea, and Amhara and Afar^29,30^. It covers an area of 54,593 km^2^ with an elevation ranging from 500 to 4000 m.a.s.l. Tigray exhibits agroecological diversity, characterized by variations in temperature, rainfall, topography, soil characteristics, vegetation cover and other natural resources^31,32^. The region is characterized by lowlands (< 1500 m.a.s.l.), which account for 53% of the total area. Midland (1500–2500 m.a.s.l.) and highland areas (> 2300 m.a.s.l.) account for 39% and 8% of the region, respectively^33^. The lowlands are characterized by high temperatures, while the highlands are by low temperatures^31^. The region has an average temperature of 18 °C^30,34^. Rainfall in the region is highly seasonal, with the majority of precipitation falling between June and September^32,35^, received within 50–60 effective rainy days^29^. The average rainfall ranges from about 200 mm in the northeast to over 1000 mm in the south western highlands^30^. The land use types of Tigray can be broadly categorized into bare land, agricultural land, forested land, bushland, shrubland, grassland, and arable land^36^. Similarly, major soil types in Tigray include cambisols, lithosols, acrisols, fluvisols, luvisols, regosols, and vertisols, which are characterized by a range of soil textures from light to heavy^29^.

Due to its natural landscape and diversified agro-ecology, Tigray is home to a rich biodiversity^37^. *H*. *forskaolii* (Fig. 1) is one of the most important bee plant species widely found in the region^5,18,21^. This plant belongs to the family *Acanthaceae.* It is a drought-tolerant perennial herb reaching a height of up to 1 m. It produces tiny white flowers with abundant pollen and nectar, its peak flowering period extends from September to November^5,18^.

### Species occurrence data

**Figure 2:**
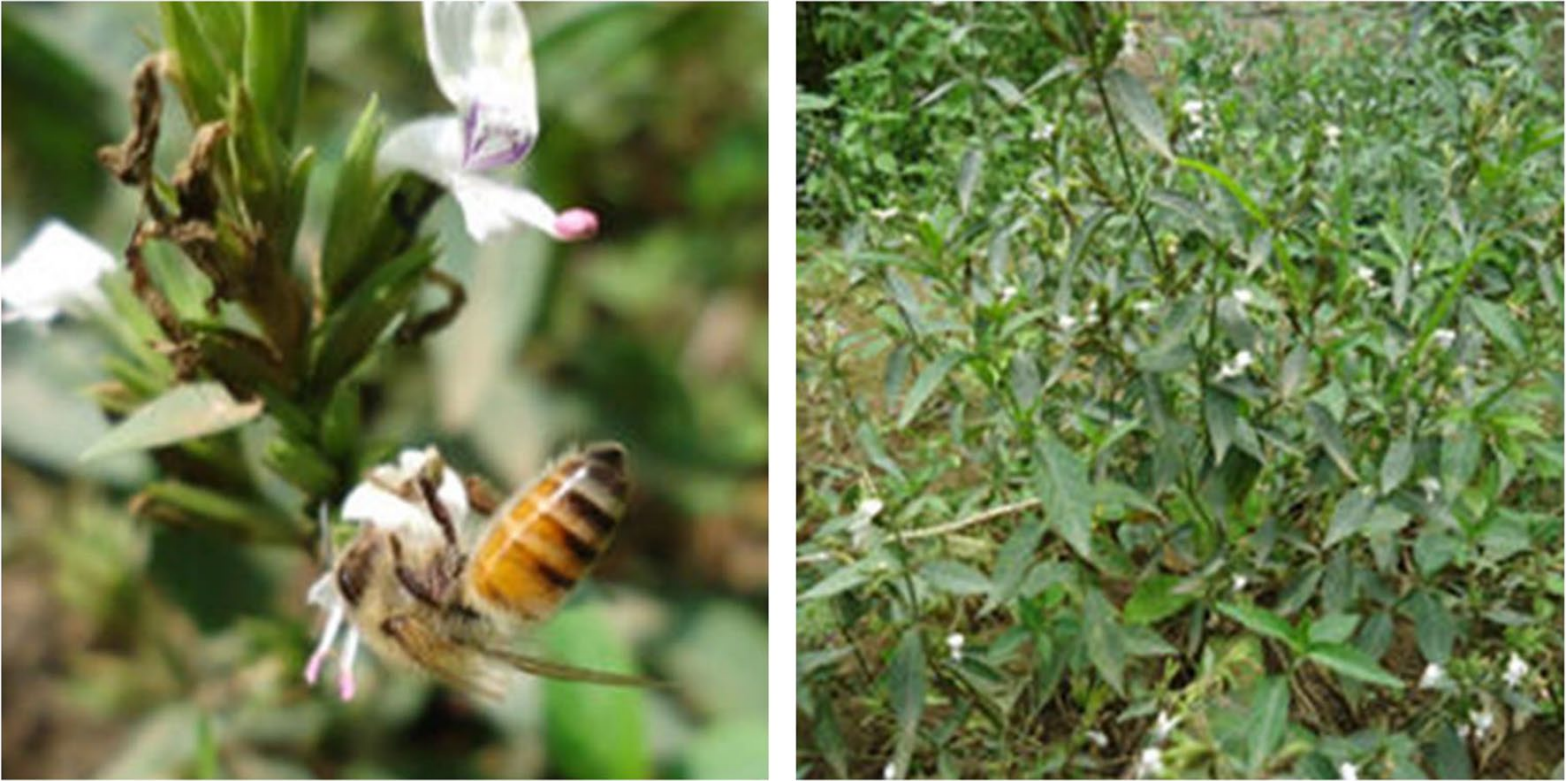
Matured *H. forskaolii* at the time of flowering (right panel) and when honeybees collecting nectar (left panel) from *H. forskaolii* in natural habitat (source^21^)*.*

Species occurrence data was collected from June to September 2020 through field surveys and discussions with key informants including bee technicians, bee experts, and beekeepers (3–5) in each village (locally *Tabia*) and district of the region. Once the presence of *H. forskaolii* was confirmed in a particular Tabia, one occurrence record with GPS coordinates was documented. To ensure comprehensive representation, each sampling point was expanded by ten additional points, following the methodology outlined by Gebru et al.^38^. To eliminate duplicated occurrence points and minimize the potential influence of environmental variations on model accuracy, we used the species distribution model (SDM) Toolbox, a Python based ArcGIS toolbox. The data were filtered to retain only one data point per grid, each 1 km by 1 km dimensions. This filtering process resulted in a total of 317 occurrence records for *H. forskaolii* (Fig. 2).

### Environmental and bioclimatic variables

To predict the distribution of *H. forskaolii* across different areas, various factors including bioclimatic conditions (temperature and precipitation), soil conditions, topography and land use types were considered (Table 1). Data related to elevation and bioclimatic variables (bio1–bio19) that represent the near-current climatic conditions (1970–2000) were obtained from the WorldClim database (http://www.worldclim.org/) at a high spatial resolution, 30 arc seconds (≈ 1 km^2^). To forecast future climatically suitable areas for *H. forskaolii*, projections from the HadGEM3-GC31-LL model were chosen due to its optimal performance in Ethiopian environments 39. Here, three scenarios of Shared Socio-economic Pathways (SSP) (ssp 126, ssp 245 and ssp 585) were selected across four time periods including the 2030s (2020–2040), 2050s (2041–2060), 2070s (2061–2080), and 2090s (2081–2100). The slope and aspect data were extracted from the Shuttle Radar Topography Mission global elevation (SRTM) (SRTM | Earthdata (nasa.gov)) using the surface analysis extension in ArcMap (version 10.4).

**Table 1:** List of variables considered in predicting the distribution of *H. forskaolii*: bioclimatic variables (temperature and precipitation), topography, land use and soil conditions.

Data type	Variable code	Variable type	Unit
Temperature	bio1	Annual mean temperature	°C
	bio2	Mean diurnal range (mean of monthly (max temp–min temp))	°C
	bio3	Iso-thermality (BIO1/BIO7) × 100	°C
	bio4	Temperature seasonality (co-efficient of variation)	°C
	bio5	Max temperature of warmest period	°C
	bio6	Min temperature of coldest period	°C
	bio7	Temperature annual range (BIO5–BIO6)	°C
	bio8	Mean temperature of wettest quarter	°C
	bio9	Mean temperature of driest quarter	°C
	bio10	Mean temperature of warmest quarter	°C
	bio11	Mean temperature of coldest quarter	°C
Precipitation	bio12	Annual precipitation	mm
	bio13	Precipitation of wettest period	mm
	bio14	Precipitation of driest period	mm
	bio15	Precipitation seasonality (co-efficient of variation)	mm
	bio16	Precipitation of wettest quarter	mm
	bio17	Precipitation of driest quarter	mm
	bio18	Precipitation of warmest quarter	mm
	bio19	Precipitation of coldest quarter	mm
Topography and land use	aez	Agroe-cological zone	code
	Landuse	Land uses	code
	Aspect	Aspect	degree
	Elevation	Elevation	meter
	Slope	Slope	Percent
Soil conditions	Bulk density	Soil bulk density	g cm^-3^
	Depth	Soil depth	cm
	OC	Soil organic carbon	g/100 g
	pH	Soil pH	code
	Texture	Soil texture	code
	Water capacity	Soil water capacity	Soil/g

Additionally, soil conditions and land use land cover data were obtained from the ISRIC World Soil Information (ISRIC Soil Data Hub | ISRIC) and Land Cover CCI—LC (Geoportail UCL-Geomatics), respectively. Given that agro-ecology also plays a crucial role in a species’ survival and habitat distribution, it has been included as an input variable in the model. There are several agro-ecological zonation systems that categorize the landscapes of Ethiopia^31,40–43^. Among these, the traditional method of climate classification which primarily relies on elevation and temperature^31,43^ has been widely used by policymakers and practitioners when formulating development strategies, such as agricultural land-use and natural resource management in Ethiopia^40^. There is also a strong correlation between altitude and agro-climatic zones^31^. Therefore, the traditional agro-ecological classification system has been employed in this study^41^ to allow policymakers and practitioners to easily understand and apply the research output. The incorporation of these diverse environmental variables ensures a comprehensive and holistic approach to climate projections.

### Variable selection procedure

**Figure 3:**
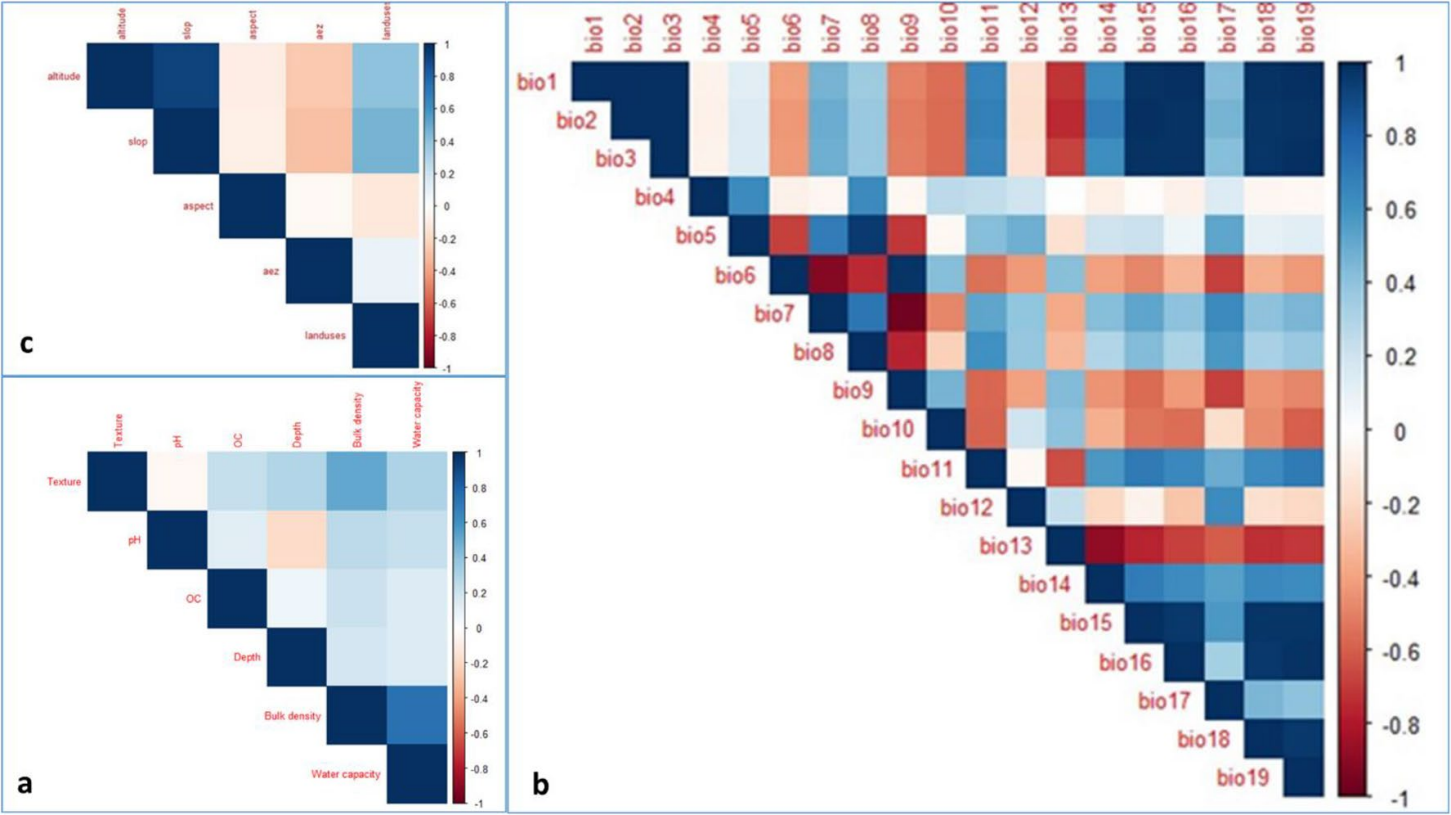
The correlation analysis of environmental variables (heatmap). The letters a, b and c represent, soil conditions, bioclimatic conditions and other factors, respectively. The rectangle with dark blue and red color, respectively shows strong a correlation between the two environmental variables (r >|0.8|). The variables mentioned, namely, bio1, bio2, bio3, bio4, bio5, bio6, bio7, bio8, bio9, bio10, bio11, bio12, bio13, bio14, bio15, bio16, bio17, bio18, bio19, aze and OC and pH correspond to Annual mean temperature, Mean diurnal range (mean of monthly (max temp–min temp)), Iso-thermality (BIO1/BIO7) × 100, Temperature Seasonality (Co-efficient of Variation), Max Temperature of Warmest Period, Min Temperature of Coldest Period, Temperature Annual Range (BIO5-BIO6), Mean Temperature of Wettest Quarter, Mean Temperature of Driest Quarter Mean Temperature of Warmest Quarter, Mean Temperature of Coldest Quarter, Annual Precipitation, Precipitation of Wettest Period, Precipitation of Driest Period, Precipitation Seasonality (Co-efficient of Variation), Precipitation of Wettest Quarter, Precipitation of Driest Quarter, Precipitation of Warmest Quarter, Precipitation of Coldest Quarter, Agroecological zone, Soil organic carbon, and Soil pH respectively.

Various techniques, including Spearman correlation, Principal Component Analysis (PCA) and the 'MaxentVariableSelection', have been used for environmental variable selection. To reduce multicollinearity among predictor variables and prevent overfitting in the model, pairwise correlation analyses were conducted in R, employing the corrplot package. Highly correlated variables, with a Spearman correlation coefficient |R|≥ 0.8, were subsequently removed (Fig. 3). To further refine our variable selection, PCA was performed to evaluate the contribution and relationship of each variable within their respective groups of climatic, soil, topography and land use using the Stats R package. The variable with the highest contribution was chosen based on its eigenvalues and eigenvectors (Table 2)^44^. Finally, we simultaneously evaluated all variables using the R package for Maxent Variable Selection (MVS)^38^. These selection techniques helped us to identify 19 environmental variables from the initial pool of 30 variables. Then, the exclusion of variables with a zero-contribution rate (bio10 and bio11) to the model's prediction was performed to improve its performance. Finally, seven variables (bio4, bio8, bio9, bio17, aez, land use, texture) were selected for predicting the distribution of *H. forskaolii* using MaExent (Table 3).

**Table 2:** Principal component analysis (PCA) results of 19 bioclimatic variables.

Variables cod	Variable type	PCA1	PCA2	PCA3	PCA4	PCA5
bio1	Annual mean temperature	0.93	− 0.32	0.05	− 0.01	0.19
bio2	Mean diurnal range (mean of monthly (max temp–min temp))	0.03	0.77	− 0.20	0.31	0.44
bio3	Iso-thermality (BIO1/BIO7) × 100	− 0.79	0.19	− 0.08	− 0.34	0.42
bio4	Temperature seasonality (co-efficient of variation)	0.73	− 0.14	0.00	0.36	− 0.49
bio5	Max temperature of warmest period	0.96	− 0.21	0.06	0.08	0.15
bio6	Min temperature of coldest period	0.89	− 0.40	0.10	− 0.07	0.16
bio7	Temperature annual range (BIO5–BIO6)	0.65	0.48	− 0.10	0.52	0.03
bio8	Mean temperature of wettest quarter	0.90	− 0.34	0.11	0.09	0.20
bio9	Mean temperature of driest quarter	0.92	− 0.32	0.08	− 0.08	0.15
bio10	Mean temperature of warmest quarter	0.94	− 0.30	0.04	0.02	0.13
bio11	Mean temperature of coldest quarter	0.92	− 0.30	0.05	− 0.05	0.22
bio12	Annual precipitation	0.03	0.48	0.85	− 0.09	0.00
bio13	Precipitation of wettest period	0.40	0.83	0.27	− 0.11	− 0.05
bio14	Precipitation of driest period	− 0.68	− 0.60	0.30	0.09	0.08
bio15	Precipitation seasonality (co-efficient of variation)	0.70	0.55	− 0.37	− 0.14	− 0.02
bio16	Precipitation of wettest quarter	0.60	0.70	0.31	− 0.19	− 0.03
bio17	Precipitation of driest quarter	− 0.74	− 0.56	0.31	0.11	0.04
bio18	Precipitation of warmest quarter	− 0.60	0.17	0.36	0.51	0.19
bio19	Precipitation of coldest quarter	0.79	0.09	0.19	− 0.19	− 0.25
Contribution rate (%)		55.62	21.13	7.77	5.40	4.88
Cumulative contribution rate (%)		55.62	76.75	84.52	89.92	94.8

### Selection of model parameters

The prediction of the MaxEnt model can be influenced by model parameters such as feature class (FC) and beta-multiplier (BM)^38^. To enhance the accuracy of species distribution predictions, adjustments were made to the feature combination and regularization multiplier using the Enmeval package. The selection of the best FC and BM combinations was based on the least Akaike Information Criterion (AICc) values^45–47^ using the best features (linear) and regulation multiplier (RM = 3), resulting in 19 combinations. In addition, the MaxEnt iteration was set to 5000, allowing for a more comprehensive analysis. To address sampling bias, a bias file was also included in the model run.

### Model calibration and evaluation

The model’s accuracy is significantly improved when the training dataset includes a broader geographic area rather than relying solely on specific data^48^. To enhance accuracy and address uncertainty, we employed the tenfold cross-validation method, running it 10 times to minimize and average the results. For model calibration and evaluation, the species occurrence data were randomly split into a training sample (75% of total occurrences) and a test sample (25% of total occurrences)^49^. In this study, despite the potential loss of statistical independence between training and test data, the "bootstrap" approach was employed as a sampling technique^48,50^. The bootstrap method enabled MaxEnt to replace the entire population data with the data used for model development.

The model’s performance was evaluated using Area Under the Curve (AUC) and the True Skill Statistic (TSS) metrics. AUC measures how effectively a model distinguishes between presences and absences, with its value ranging from 0 to 1. A value of 1 indicates a perfect fit of the model. TSS evaluates a model’s ability to detect true presence (sensitivity) and true absence (specificity), calculated as sensitivity + specificity − 1 (Eq. 1). The TSS value, ranging from − 1 to 1, signifies the degree of agreement between predicted and actual points, with a score of 1 indicating perfect agreement^51,52^.

1 $$TSS = \frac{(TP \times TN - FP \times FN)}{{(TP + FN) \times (FP + TN)}} = Sensitivity + Specificity - 1$$

where: TP (True Positive), TN (True Negative), FP (False Positive) and FN (False Negative).

### Habitat suitability classification

MaxEnt uses a habitat suitability index (HSI) ranging from 0 to 1 to evaluate the habitat's suitability for a species, considering diverse environmental variables. A value of 1 represents the highest suitability for the habitat^53^. Here, the generated habitat suitability map was categorized into four groups: not suitable, less suitable, moderately suitable, and highly habitats using the Natural Breaks method in ArcGIS and the area within each category was calculated (Fig. 11; Table 4).

## Results

### MaxEnt model performance evaluation

**Figure 4:**
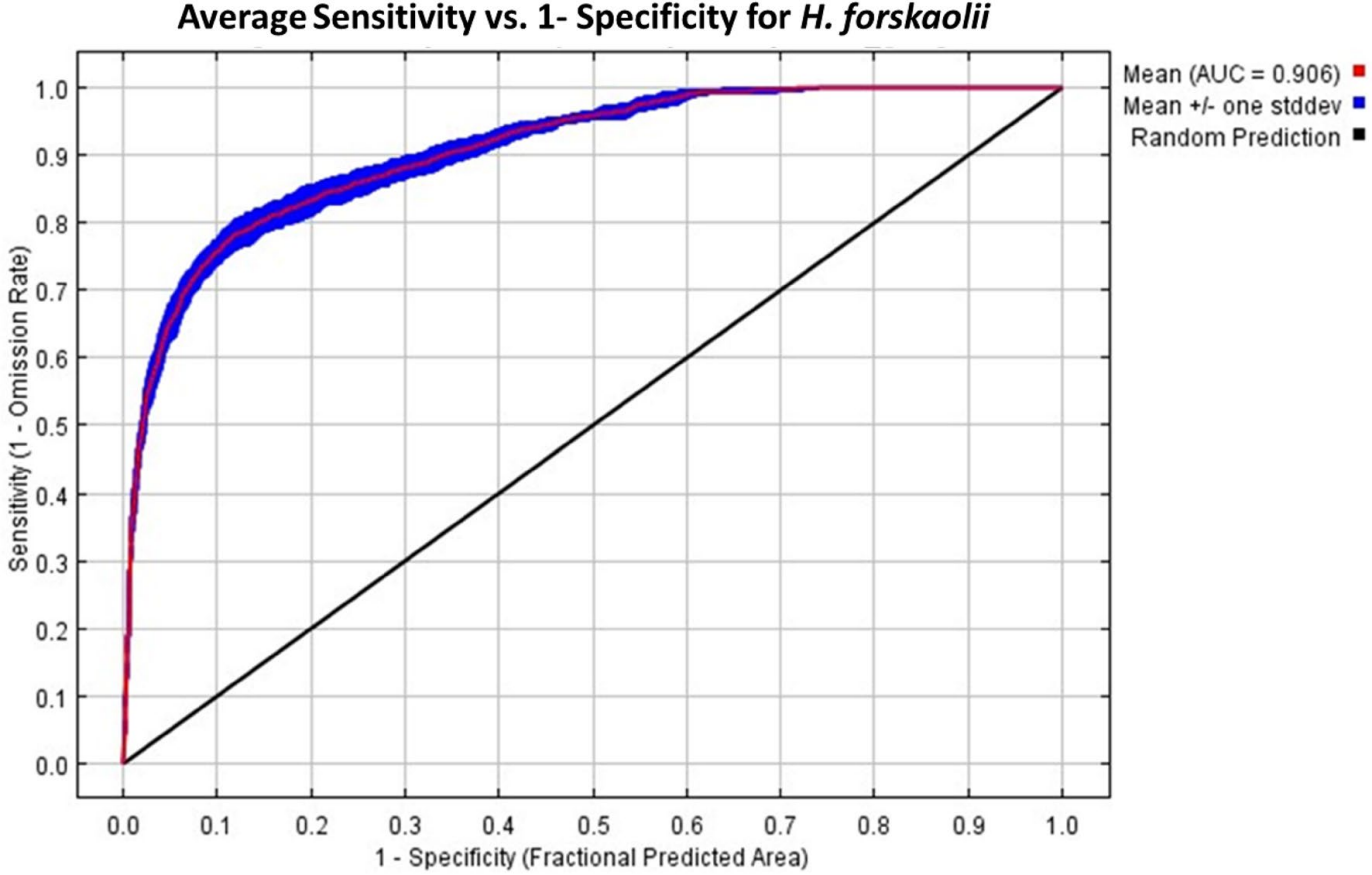
ROC (Receiver Operating Characteristic) curves and AUC (Area Under the Curve) averages for MaxEnt with 10 repeated runs.

The average area under the curve (AUC) value, based on ten repetitions of the model, is 0.906 (Fig. 4) indicating excellent predictive ability for identifying potentially suitable habitats for *H. forskaolii*. Additionally, the True Skill Statistic (TSS) value is 0.634, demonstrating the model’s good discriminating power between points with and without the presence of *H. forskaolii.* Overall, the results suggested that the final model developed for predicting suitable habitats for *H. forskaolii* in Tigray is highly accurate, indicating its effectiveness as observed in the model performance evaluation.

### Contribution of bioclimatic variables to habitat suitability

**Figure 5:**
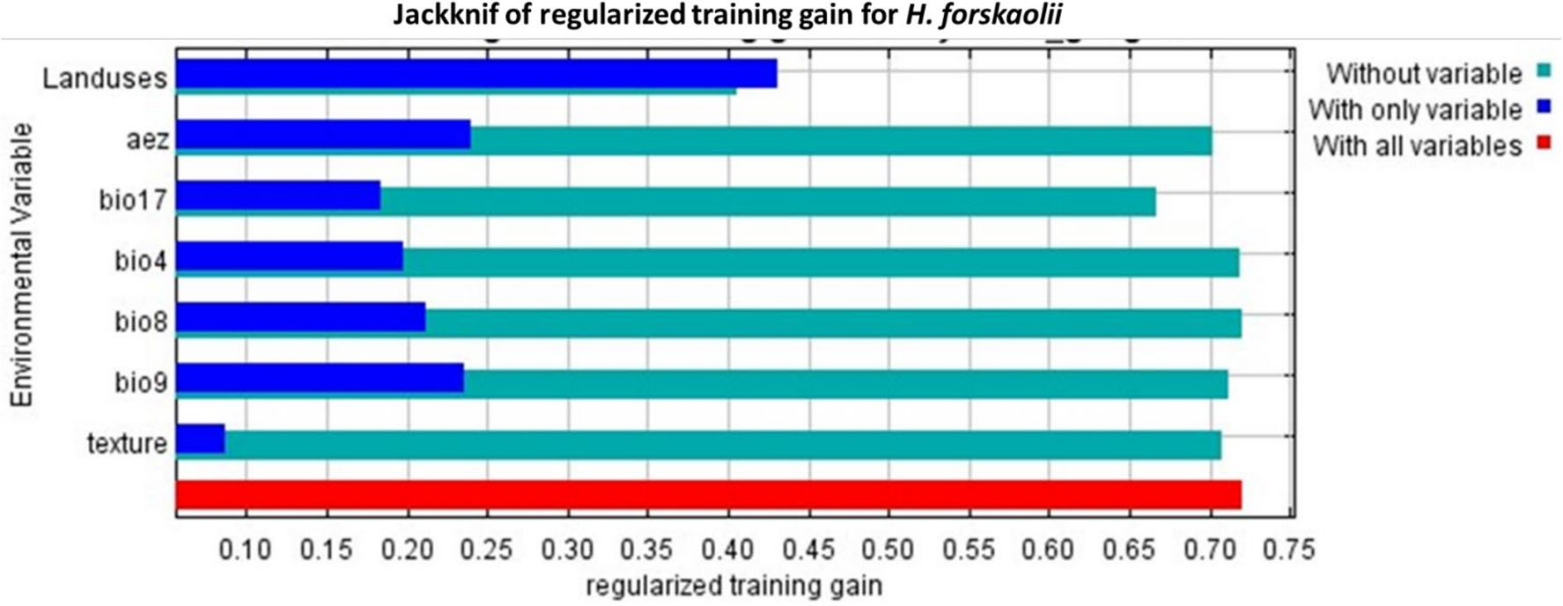
The contribution of environmental factors as determined by the Jackknife analysis of regularized training gain in MaxEnt models for *H. forskaolii.* The variables mentioned, namely aez, bio17, bio4, bio8, and bio9 correspond to the Agroecological zone, Precipitation of the Driest Quarter, Temperature Seasonality (Co-efficient of Variation), Mean Temperature of Wettest Quarter and Mean Temperature of the Driest Quarter, respectively.

The relative importance of predictor variables and their contributions were evaluated using the Jackknife method (Table 3; Fig. 5). The land use, agro-ecology, and precipitation of the Driest Quarter (mm) (bio17) are identified as crucial factors influencing the distributions of *H. forskaolii* (Table 3), collectively contributing to 89.3% of the model's predictive power (Table 3). Furthermore, agro-ecology and land use have shown the highest regularized training gains (Fig. 5). These results strongly indicate that land use and agro-ecology are the most important determinants influencing the occurrence of *H. forskaolii*. Conversely, the contribution of environmental factors such as the mean temperature of the Driest Quarter (bio9), temperature seasonality (bio4), and mean temperature of the Wettest Quarter (bio8) was small, accounting for 4.6% (Table 3).

**Table 3:** Relative contribution of environmental variables against existing climatic conditions as explained by the Maxent model.

Variable cod	Variable type	Percent contribution	Permutation importance (%)
Land uses	Land uses	50.1	8.1
aez	Agroecological zone	28	2.9
bio17	Precipitation of driest quarter	11.2	31.4
texture	Texture	6.1	8.1
bio9	Mean temperature of driest quarter	3.9	40.1
bio4	Temperature seasonality (co-efficient of variation)	0.5	3.8
bio8	Mean temperature of wettest quarter	0.2	5.6

### Environmental variable response curves

**Figure 6:**
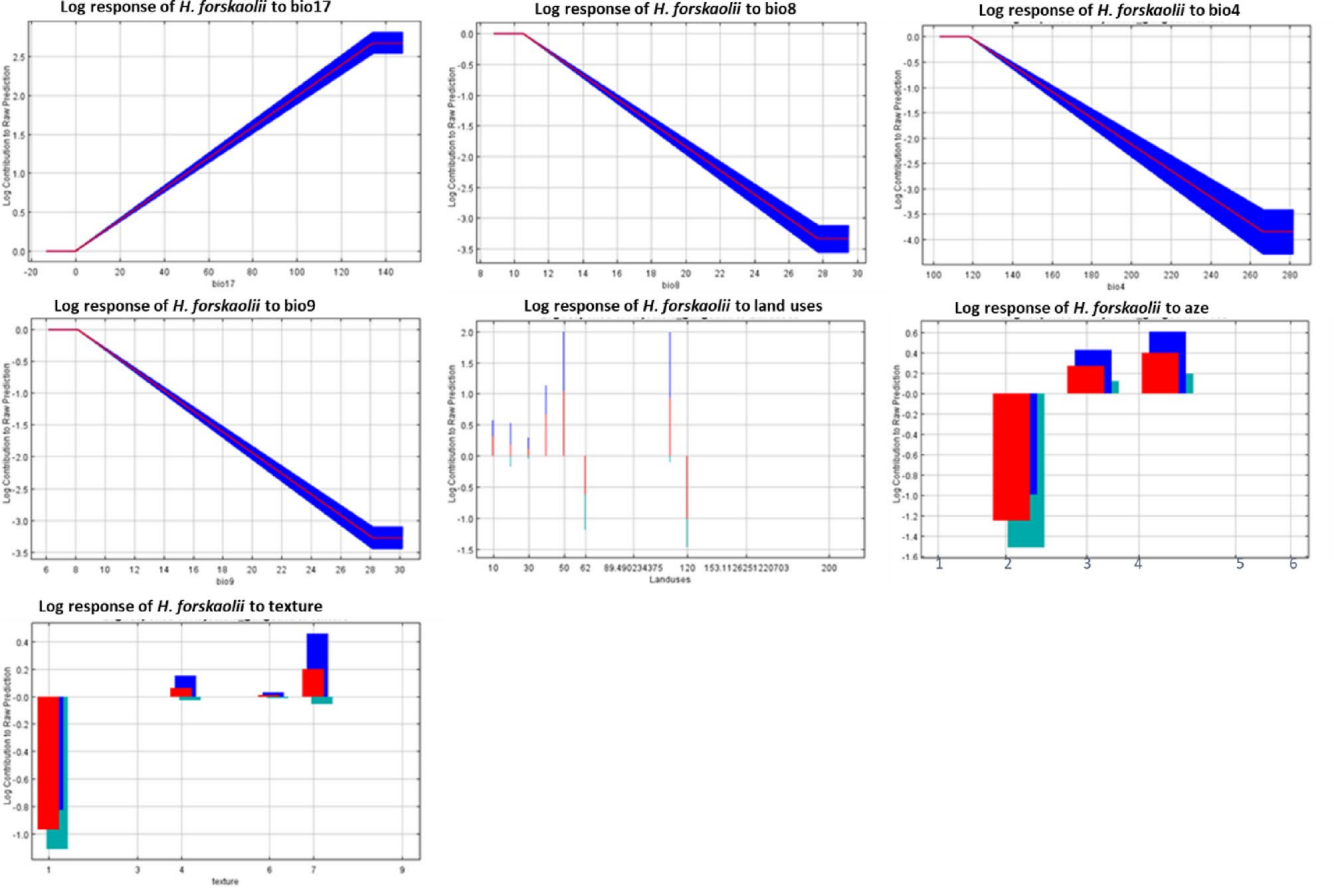
The relationship of major environmental factors with habitat suitability for *H. forskaolii.* The variables mentioned, namely bio4, bio8, bio9, and aez correspond to Temperature Seasonality (Co-efficient of Variation), Mean Temperature of the Wettest Quarter, Mean Temperature of the Driest Quarter and Agroecological zone, respectively. The areas under the response curve for agro-ecology, agroecological zones (AEZ 1 to 6), and land uses (10 to 120) are detailed in the legends of Figs. 7c and 8a.

**Figure 7:**
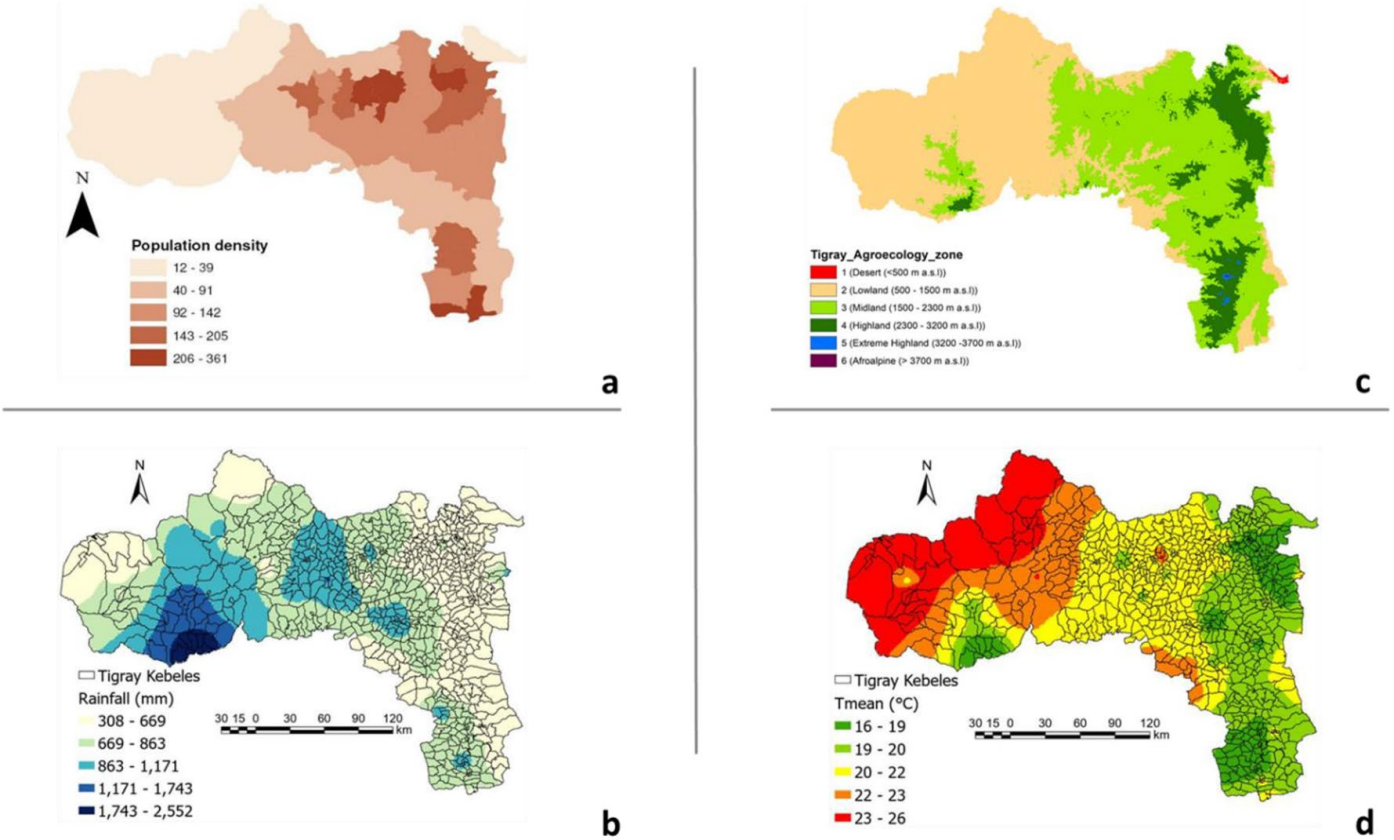
Honey bee population density (**a**), annual precipitation (**b**) and Agroecological zonation (**c**) and mean annual temperature (**d**) in Tigray*.* To determine the colony population density, we have used average data (2004–2021) from the Central Statistical Agency of Ethiopia (CSA). The honey bee population density is expressed as the average number of honey bee colony population per square kilometer. Figures were created using ArcMap (version 10.4).

**Figure 8:**
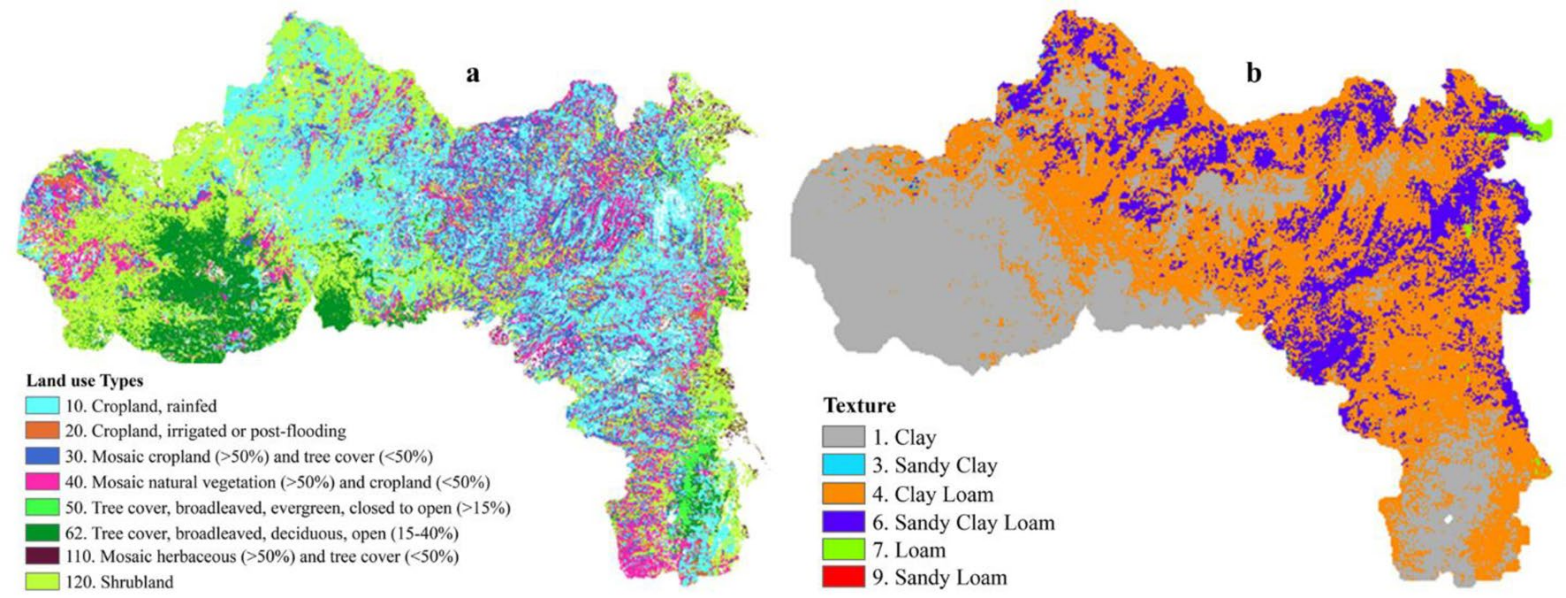
Land use types (**a**) and Soil texture (**b**) that influence the distribution of *H. forskaolii* in Tigray. The numbers in the legend, ranging from 10 to 120 in the land use classification (i.e., based on the Land Cover CCI (Geoportail UCL-Geomatics) and from 1 to 9 for soil texture types, respectively used for modeling the species distribution and produce their response curves. Figures were created using ArcMap (version 10.4).

Understanding the climatic thresholds for the core habitats of *H. forskaolii* is crucial for effective land management and natural resource conservation efforts. Identification of these thresholds has provided insights into the suitability of the specific environmental conditions for the growth and distribution of the species. According to the response curve for land use, the natural suitable habitat for *H. forskaolii* mainly includes areas dominated by broad leaved evergreen trees followed by mosic herbaceous cover (Figs. 6, 7 and 8a). Whereas, areas covered with shrubs and deciduous trees that are open, covering between 15 to 40% of the land areas are less suitable habitat for the distribution of *H. forskaolii*. The response curve for the precipitation of the Driest Quarter (bio17) indicates that the likelihood of *H. forskaolii* occurrence increases with precipitation during the driest quarter and reaches its peak when the area receives about 130 mm. This suggests the importance of a certain amount of precipitation during the driest season for the survival of the plant. The plant species is commonly found in core areas with specific soil texture classes such as loam, clay loam and sandy clay loam soils (Fig. 8b). This is supported by the response curve for soil texture, which showed that the occurrence likelihood of *H. forskaolii* peaks when the soil type is loamy sand, followed by sandy loam (Fig. 8b). Additionally, the response curve for the mean temperature of the wettest quarter (bio8) and mean temperature of the driest quarter (bio9) indicated that the occurrence likelihood of *H. forskaolii* reaches its peak when the temperature falls below 11 °C and 8 °C, respectively. The study also revealed the highest habitat suitability in the highland and mid-highland agroecological zones compared to the lowlands.

### Current habitat distribution of *H. forskaolii*

**Figure 9:**
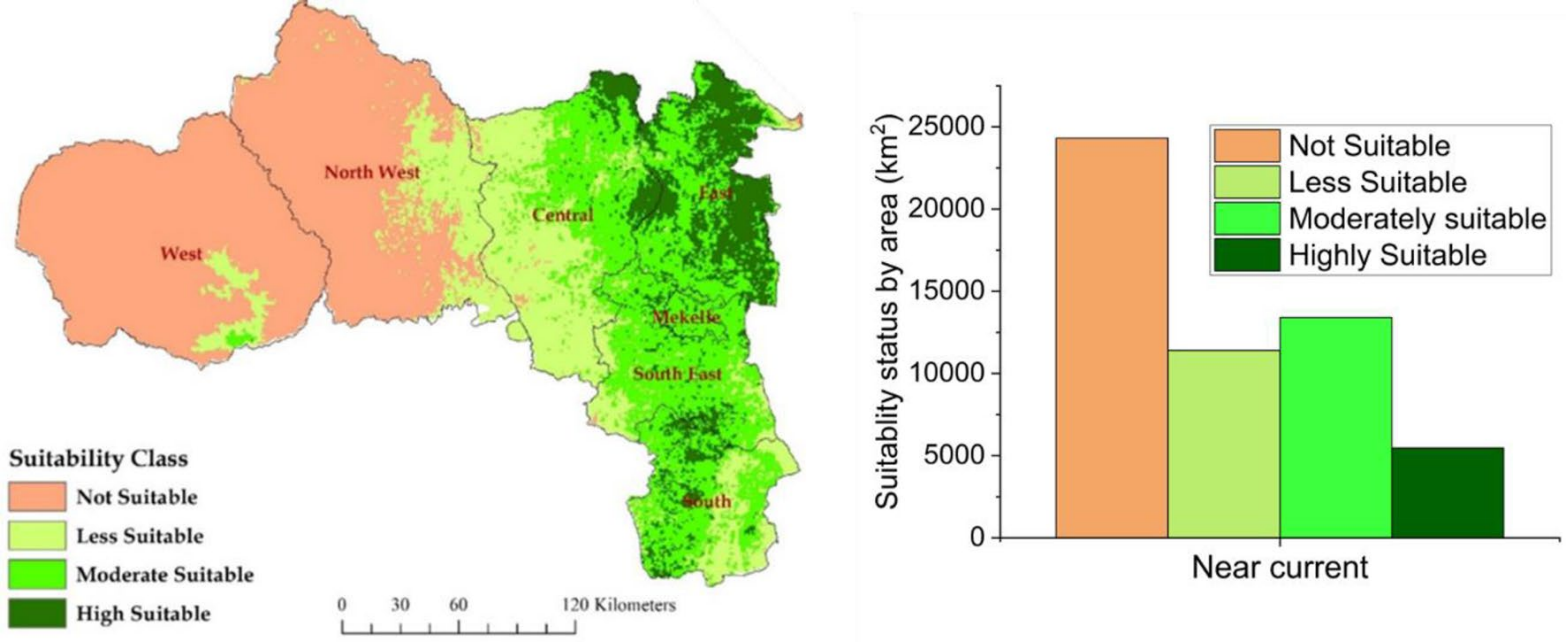
Current habitat distribution of *H. forskaolii (*left panel) and its suitability status (right panel) in Tigray. Figures were created using ArcMap (version 10.4).

Under current climatic conditions, about 55.5% (30,272 km^2^) of Tigray's land mass is identified as climatically suitable habitat for *H. forskaolii* (Fig. 9). These suitable habitats are mainly concentrated in the highlands and mid-highlands of the eastern and southern zones. Among the suitable areas, a significant portion was classified as highly (10%, 5467 km^2^) and moderately (24.6%, 13,407 km^2^) suitable habitats for *H. forskaolii*. These suitable habitats provide optimal conditions for the growth and distribution of the species. Most of the eastern and southern zones of Tigray are classified as moderately and highly suitable habitats (Fig. 7c). Conversely, areas less suitable for *H. forskaolii,* are primarily located in the central and northwest zones, covering 20.9% (11,398 km^2^) of the region. On the other hand, large parts of western and northwestern Tigray, characterized by lowlands (Fig. 7c), are dominantly not suitable for the growth and distribution of *H. forskaolii.* Identifying the suitable and unsuitable habitats for *H. forskaolii* has important implications for the management of the species including its conservation and cultivation. The concentration of moderately and highly suitable areas in the eastern and southern zones suggests that these areas should be prioritized for the species conservation and the white specialty honey production. In addition, honey bee population density has followed the same spatial pattern (Fig. 7a) as that of *H. forskaolii* distribution in Tigray. While *H. forskaolii* likely contributes significantly to this overlap, it may not entirely account for it since other species of bee forage plants in the area could also contribute to the high honey bee population density and suitability areas for beekeeping^17,18^. As *H. forskaolii* thrives in these areas, it is crucial to protect and preserve its natural habitats to ensure the long-term survival of the species and enhance white honey production in the region.

### Future habitat distributions of *H. forskaolii*

**Figure 10:**
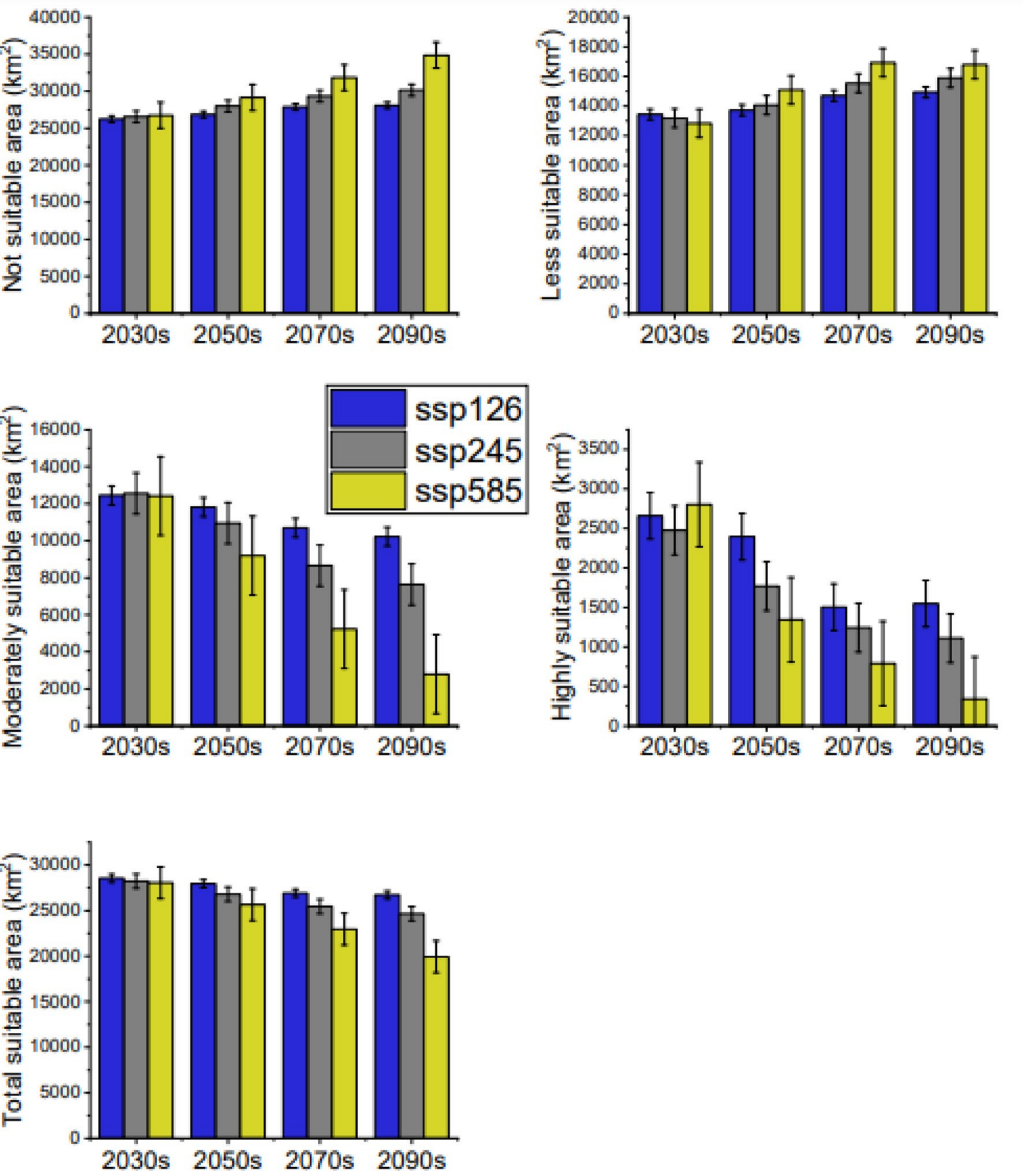
Future (2030s, 2050s, 2070s, and 2090s) habitat suitability: not suitable, less suitable, moderately suitable and highly suitable under three climate scenarios: ssp126, ssp245 and ssp585.

**Figure 11:**
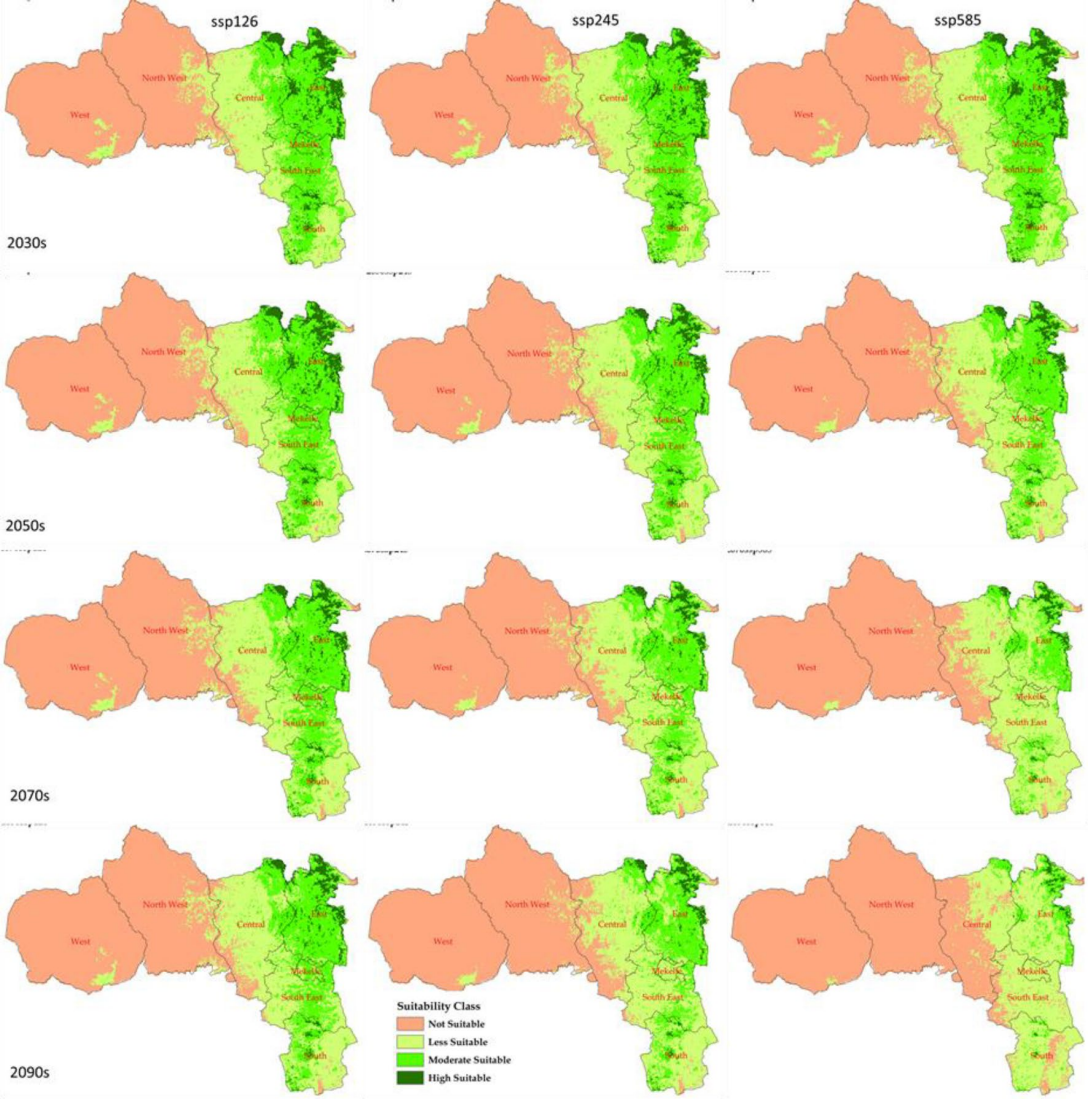
The future spatial habitat distribution of *H. forskaolii* in Tigray, under climate scenarios of SSP126, SSP245 and SSP585 during the 2030s, 2050s, 2070s, and 2090s. Figures were created using ArcMap (version 10.4).

Table 4 depicts future *H. forskaolii* ’s species distribution predicted under three future climate change scenarios (ssp126, ssp245 and ssp585). Under all these scenarios, the predicted suitable habitat for *H. forskaolii* is expected to decrease across the four time periods included in this study: 2030s, 2050s, 2070s, and 2090 (Figs. 10 and 11). However, the suitable habitat for *H. forskaolii* is expected to decrease significantly under the ssp585 scenario and decreases exponentially with time. Under this scenario, the suitable habitat for *H. forskaolii* will decrease by 4.26%, 8.67%, 13.56%, and 19.09% compared to the current distribution in the 2030s, 2050s, 2070s and 2090s, respectively (Fig. 10).

**Table 4:** Projected suitable areas of *H. forskaolii* under current (1970–2000) and future climate scenarios (ssp126, ssp245 and ssp585) over four time periods (2030s, 2050s, 2070s and 2090s) in Tigray.

Scenarios	Not suitable (km^2^)	Less suitable (km^2^)	Moderately suitable (km^2^)	Highly suitable (km^2^)	Total suitable area (km^2^)	Suitable area percentage change (%)
Current	24,318	11,398	13,407	5467	30,272	–
2030ssp126	26,248	13,437	12,445	2660	28,542	− 3.36
2030ssp245	26,572	13,184	12,559	2475	28,218	− 3.95
2030ssp585	26,741	12,830	12,417	2802	28,049	− 4.26
2050ssp126	26,854	13,722	11,818	2396	27,936	− 4.47
2050ssp245	28,005	14,067	10,949	1769	26,785	− 6.57
2050ssp585	29,157	15,090	9,199	1344	25,633	− 8.67
2070ssp126	27,889	14,698	10,699	1504	26,901	− 6.36
2070ssp245	29,346	15,542	8,659	1243	25,444	− 9.01
2070ssp585	31,838	16,930	5,232	790	22,952	− 13.56
2090ssp126	28,084	14,934	10,224	1548	26,706	− 6.71
2090ssp245	30,142	15,895	7,642	1111	24,648	− 10.47
2090ssp585	34,866	16,792	2,792	340	19,924	− 19.09

In addition, a significant reduction in the suitable habitat for *H. forskaolii* is expected towards the end of the century under the ssp245 scenario. The result revealed that suitable habitat for *H. forskaolii* under the ssp245 scenario is predicted to reduce by 9.01% and 10.47% by the 2070s and 2090s, respectively (Fig. 11). Similarly, under the ssp126 scenario, the suitable habitat for *H. forskaolii* is expected to decrease progressively from 3.36% in the 2030s to 6.71% in the 2090s.

Under ssp585 and ssp245 scenarios areas regarded as not suitable and less suitable habitats for *H. forskaolii* are expected to increase, particularly in the northwestern and central zones of Tigray (Figs. 10, 11). Conversely, areas considered moderately and highly suitable habitats for *H. forskaolii* are expected to decrease, with the most significant reduction anticipated under ssp585 scenarios towards the end of the century. Consequently, the areas that are highly suitable habitats for *H. forskaolii* will be limited to a few spots in the eastern part of the region (Fig. 11).

## Discussion

Climate change significantly affects the distribution, growth, and survival of many indigenous plant species, which are primary food sources (pollen and nectar) for honey bees^26–28^. Therefore, understanding how these plants respond to global climate change is crucial for understanding and managing their habitat distribution. *H. forskaolii* is one of the most important honey bee plants in Ethiopia’s Tigray region, producing abundant pollen and nectar for honey bees^5^*.* However, due to the decline in its population distribution, further investigation into the contributing environmental factors is necessary to develop possible conservation and restoration strategies. Thus, predicting the current and future distribution of *H. forskaolii* under various climate change scenarios is essential for informed policymaking.

### *H. forskaolii* species habitat distribution using MaxEnt model

The model's predictive performance was evaluated using AUC, with values ranging from 0 to 1. A higher AUC value indicates better prediction performance^54^. More specifically, an AUC value greater than 0.9 indicates highly accurate prediction^55^. In this study, an average AUC value of 0.906 was obtained indicating that the simulation effect was excellent and, thus, the model can be used to predict the potential distribution of *H. forskaolii.* The result also reflected a high level of discrimination between suitable and unsuitable habitats^56,57^. The TSS values range from 0 to 1, with values close to 1 indicating higher model accuracy^52^. The TSS values were 0.634, suggesting a high probability of accurate prediction^54^, indicating the model's effectiveness in discriminating between the presence and absence of *H. forskaolii s*pecies^52,54^. Overall, the results indicated that the final model developed for predicting suitable habitats for *H. forskaolii* in Tigray is highly reliable and accurate.

### Determinant factors for *H. forskaolii* distribution

Under the current climatic conditions, 55.5% of Tigray's land area (30,272 km^2^) is a suitable habitat for the growth and distribution of *H. forskaolii*. Moderately and highly suitable areas for *H. forskaolii* are concentrated in the eastern, central, south, and southeast zones, which are characterized by highland and mid-highland agroecological zones (Fig. 7c). The prevalence of suitable habitats in these zones aligns with the abundance of *H. forskaolii*. Notably, these areas are well known for their white honey production, primarily produced from this plant species^15^. Conversely, suitability is lower in the hot lowland agroecological zones in the northwest and west zones (Fig. 9)^31^. These results suggest that the most favorable environmental conditions for the plant’s distribution are found in the highland to mid-highland areas of Tigray.

Moreover, the occurrence of *H. forskaolii* is found to be significantly influenced by agro-ecology (Table 3), where habitat suitability is highest in the highland and mid-highland areas. These areas are characterized by lower temperatures (Fig. 7) as compared to the lowland areas in Tigray^31^. The response curve for the mean temperature of the wettest quarter (bio8) and driest quarter (bio9) aligns with these results. These results indicate that the likelihood of *H. forskaolii* incidence reaches its peak when the temperature falls below 11 °C for bio8 and 8 °C for bio9. This means that colder environments are more conducive, demonstrating the significance of temperatures in influencing the distribution of *H. forskaolii.*

The prevalence of suitable habitats in the highland and mid-highland agroecological zones (Fig. 9), along with the crucial role of agro-ecology in predicting the plant’s distribution (Table 3), suggests a potential association between preferred habitat, agro-ecology, and altitude. Thus, agro-ecology, a specific geographical area with certain environmental conditions, such as climate, soil, topography, and hydrology^58^ has been a key factor in determining habitat suitability for *H. forskaolii.* However, the specific relationship between climate and ecological variables may vary depending on the species and environmental conditions^59^. The overall findings underscore the agroecological preferences of *H. forskaolii*, highlighting the necessity to prioritize specific ecological-regions in conservation and production initiatives. These suitable habitats are proposed for the conservation of *H. forskaolii* and could also be used as a source of planting materials for the less suitable areas in the region augmented with improved management.

Precipitation during the driest quarter (bio17) is a bioclimatic variable that indicates the total precipitation received during that specific quarter. It is commonly used to assess water availability and drought stress in a given area^60^. In our study, bio17 ranks among the top three environmental variables influencing the model's prediction (11.2%), signifying its relevance in determining the occurrence of the plant species. The response curve for bio17 indicated that the likelihood of *H. forskaolii* occurrence peaks between 0 and 130 mm in precipitation (Fig. 6), highlighting the importance of water availability during the driest season for the species’ survival, growth and distribution. Lower bio17 values (low precipitation during the driest quarter) may also suggest that the plant species has a higher tolerance to drought stress or water scarcity. The annual precipitation in the eastern and southeastern parts of Tigray, where *H. forskaolii* is best suited, is lower compared to the western part (Fig. 7b). These areas also receive a small amount of precipitation during the dry season^32^. Hence, the variation in suitable habitat coverage for *H. forskaolii* across different administrative zones in Tigray may also be attributed to variations in rainfall distribution.

Soil texture is the most important basic physical property of soil that determines water holding and infiltration capacity^61^ which in turn plays a significant role in shaping natural vegetation patterns^62,63^. In this regard, woody plant dominance is associated with coarse-textured soils, while fine-textured soil supports herbaceous species^63^. In our study, the soil textural class emerged as a crucial determining factor (6.1%) for the occurrence of *H. forskaolii* (Table 4). Loam, clay loam and sandy clay loam soils are suitable for *H. forskaolii* (Fig. 7b). While clay soil, which retain more water, is less suitable, especially in logged areas. Most of the plant's distribution is also found in clay loam and sandy clay loam soils (Fig. 8b). This indicates that the plant is adapted to low soil moisture content^64^. In semi-arid environments, coarse-textured soils like loam, clay loam and sandy clay allow water to infiltrate better during rainy season and plants with extensive root systems, such as *H. forskaolii*, can access this stored water during dry season^63^.

Land use plays a crucial role in shaping species distribution by directly impacting their habitats and ecosystems^65^. Changes in land use due to human activities, such as deforestation, industrial development, urbanization, and agricultural land expansion have led to declines in species abundance, diversity and ecosystem health worldwide^66,67^. The present study also identified land use as the most determinant factor influencing the distribution of *H. forskaolii*, contributing more than 50% to the model’s prediction (Table 3). In our study, land uses dominated by evergreen trees and mosaic herbaceous with good vegetation cover that create relatively cooler microclimate have been identified as the most suitable habitat for *H. forskaolii* (Fig. 6). While areas dominated by shrubland and deciduous trees that shed their leaves during the hot dry season are considered less suitable for this species. Therefore, *H. forskaolii* is a shade-tolerant plant, well-adapted to low light conditions beneath the higher canopy of large and tall trees. Consistent with this, the response curve for the Mean Temperature of the Driest Quarter (Fig. 9), shows that the suitability decreases significantly as the temperature of the quarter increases from 10 to 25 °C. These results suggest that areas with good vegetation cover provide continues shade and a relatively colder microclimates are the natural habitat for *H. forskaolii*^68^. In addition, *H. forskaolii* exhibits adaptation to a diverse range of land use types, including riverine forests, rocky slopes, open woodlands, mixed woodlands, and wooded grasslands^5^. On the other hand, grasslands and agricultural land are made up of open spaces with annual crops and shorter vegetation at which the *H. forskaolii* has not developed resistance to regular grazing and trampling effects of livestock.

However, various natural and anthropogenic activities, such as frequent drought, deforestation, land degradation, agricultural land expansion, armed conflict, and other human activities, have significantly contributed to the decline in forest cover in Tigray^36,69^. Only from 1965 to 1994, the forest land coverage in Tigray decreased by 2.9% in favor of arable land and rangeland^36^. This suggests a transformation in land use patterns over those years, highlighting a trend where forest and bush areas have been reduced to make way for increased arable cultivation and grazing lands., Between 2000 and 2020, vegetation cover in Tigray increased by 2% due to a continuous reforestation and natural resource rehabilitation initiative^70–72^. However, during the period 2020–2022, the vegetation cover decreased by 5% of the area^72^, and forest cover declined on average by 14.5% due to the increased demand for firewood and charcoal during the wartime^69^. *H. forskaolii’s* coverage and population growth could also be affected by military actions such as destroying trees, soil and water conservation efforts, disrupting soil health^69,72,73^. These suggest that the loss of *H. forskaolii* habitat is not solely an effect of climate changes but also due to other anthropogenic activities. Therefore, to revitalize and conserve the important honey bee plant, *H. forskaolii* appropriate land use policies, plantation and conservation practices should be implemented.

### Impact of climate change on honeybee plants

Climate change can have a significant impact on honey bees by disrupting the diversity and availability of plants crucial for collecting pollen and nectar. The alteration in climate patterns poses a threat to honey bees as it destroys their natural food sources, potentially leading to adverse effects on nutrition, overall health, and productivity^28^. The current study indicates a decline in the suitable habitat for *H. forskaolii,* a major honey bee plant in Tigray due to climate change, particularly under the ssp585 climate scenario (Fig. 11 and Table 4). Projections under this scenario show a gradual decline in suitable habitats, from 4.26% in the 2030s to 19.09% in the 2090s (Fig. 11 and Table 4), compared to the current distribution (Fig. 10). Similarly, the suitable habitat for *H. forskaolii* is anticipated to decrease under the ssp245 scenario. These results underscore the negative impacts of climate change on the availability of this plant species. This could exacerbate the existing shortage of honey bee forages in the region^74^. This will also significantly affect the livelihoods of the local beekeepers by reducing white honey production, a major source of income. The scarcity of bee forage may also lead to absconding, wherein bees leave their hives in search of food, ultimately reducing honey production. Therefore, the reduction in highly suitable habitats in the eastern and south-eastern, and the reduction in moderately suitable habitats in the western, southern and central zones highlights the necessity for urgent interventions aimed at preserving, sustaining, restoring and promoting the honey bee plant populations. These areas are homes for densely populated honey bees in Tigray (Fig. 7a). Acknowledging the diverse impacts of climate change across Tigray and identifying moderately and highly suitable areas highlights the importance of prioritizing specific localities for conservation efforts aimed at preserving *H. forskaolii* that would be used as a source of planting material in promoting this important species through an augmented management in wider areas.

## Conclusion

In this study, the MaxEnt model was used to identify potential habitats for *H. forskaolii* in Tigray, northern Ethiopia. Presently, about 55.5% of Tigray's total land mass is identified as suitable habitat for *H. forskaolii,* with a predominant concentration in the highlands and mid-highlands of the eastern, central, southeastern and southern zones. The key determinant factors influencing the distribution of this plant species include land use patterns, agroecological conditions, precipitation levels during the Driest Quarter (mm) (bio17) and soil texture, collectively accounting for 95.4% of the model's predictive power. *H. forskaolii* thrives in the midland to highland agro-ecological zones of the region, which are characterized by low seasonal mean temperatures, especially during the wettest and driest quarters. Moreover, habitats rich in evergreen trees and mosaic herbaceous species, and loam, clay loam and sandy clay soil types are particularly conducive to the growth of *H. Forskaolii.*

Future climate change scenarios indicate a general trend of decreasing suitable habitats for *H. forskaolii*. Predictions under the ssp585 scenario reveal particularly concerning outcomes, with a notable decline from 4.26% in the 2030s to 19.09% in the 2090s. This highlights the detrimental impact of climate change on bee forage availability and the overall well-being of honey bees*.* Moreover, the assessment of current and projected habitat suitability for *H. forskaolii,* provides valuable scientific insights for formulating adaptation and mitigation strategies, thereby enhancing the development of climate-resilient bee forages.

Therefore, research should focus on identifying and evaluating technologies to promote climate change adaptation and mitigation efforts for the *H. forskaolii* species. This includes developing resilient cultivars by investigating the physiological and genetic mechanisms that enable the species to adapt to changing climate conditions. Additionally, exploring management strategies to support *H. forskaolii*’s adaptation is crucial for effective climate change adaptation and mitigation efforts.

## Data Availability

All data generated during this study are included in the main body of this published article and its supplementary information. However, if anyone requires the raw data we used to produce the results during the current study (i.e., the environmental variables and occurrence data), it is available from the corresponding author upon reasonable request.
